# Regulation of GABA_A_ Receptors Induced by the Activation of L-Type Voltage-Gated Calcium Channels

**DOI:** 10.3390/membranes11070486

**Published:** 2021-06-29

**Authors:** María Clara Gravielle

**Affiliations:** Instituto de Investigaciones Farmacológicas (ININFA), Facultad de Farmacia y Bioquímica, Universidad de Buenos Aires, Buenos Aires C1113AAD, Argentina; graviell@ffyb.uba.ar; Tel.: +54-11-5287-4524 (ext. 25)

**Keywords:** GABA_A_ receptors, L-type voltage-gated calcium channels, transcription, phosphorylation, trafficking, clustering

## Abstract

GABA_A_ receptors are pentameric ion channels that mediate most synaptic and tonic extrasynaptic inhibitory transmissions in the central nervous system. There are multiple GABA_A_ receptor subtypes constructed from 19 different subunits in mammals that exhibit different regional and subcellular distributions and distinct pharmacological properties. Dysfunctional alterations of GABA_A_ receptors are associated with various neuropsychiatric disorders. Short- and long-term plastic changes in GABA_A_ receptors can be induced by the activation of different intracellular signaling pathways that are triggered, under physiological and pathological conditions, by calcium entering through voltage-gated calcium channels. This review discusses several mechanisms of regulation of GABA_A_ receptor function that result from the activation of L-type voltage gated calcium channels. Calcium influx via these channels activates different signaling cascades that lead to changes in GABA_A_ receptor transcription, phosphorylation, trafficking, and synaptic clustering, thus regulating the inhibitory synaptic strength. These plastic mechanisms regulate the interplay of synaptic excitation and inhibition that is crucial for the normal function of neuronal circuits.

## 1. Introduction

The activation of GABA_A_ receptors in the mammalian brain is responsible for most fast synaptic inhibition. They are members of the Cys-loop ligand-gated ion channel family, which also includes nicotinic acetylcholine, 5-hydroxytryptamine type 3, and glycine receptors. GABA_A_ receptors are homo- or hetero-pentameric ion channels comprised in mammals of different combinations of 19 subunits. Based on sequence homology, these subunits are divided into eight subunit classes, some of them with several subtypes. Six α, three β, three γ, one δ, one ε, one θ, one π, and three ρ subunits have been cloned [1]. Subunit diversity is increased by alternative splicing and promoter usage. The most abundant GABA_A_ receptor is constructed from two α1, two β2, and one γ2 subunits. However, the results from a recent report demonstrated that the β3 subunit is required for GABAergic synaptic transmission [2]. The recent discovery of transmembrane accessory proteins that modulate GABA_A_ receptor trafficking and function indicates that the receptor exists as a complex and adds an additional level of complexity to its regulatory mechanisms [3,4,5,6,7,8].

The activation of GABA_A_ receptors in mature neurons predominantly leads to hyperpolarizing and inhibitory responses. In immature neurons, however, GABA_A_ receptor responses are depolarizing and excitatory. This change in the action of GABA is due to the expression during the development of a K^+^-Cl^−^ co-transporter, KCC2, which extrudes chloride from the cells [9]. On the other hand, GABA actions can be depolarizing and excitatory in the mature brain under physiological and pathological conditions that involve intense GABA_A_ receptor activation [10,11,12,13].

Dysfunctional alterations of GABA_A_ receptors occur in numerous disorders such as epilepsy [14,15,16,17], anxiety [18], depression [19], autism [16,17], schizophrenia [20], and Alzheimer’s disease [21], among others. Different allosteric modulators of GABA_A_ receptors, including benzodiazepine site ligands, barbiturates, ethanol, neuroactive steroids, and some anesthetics, are pharmacologically important. Benzodiazepines, the most frequently prescribed drugs acting at GABA_A_ receptors, exert anxiolytic, sedative, anticonvulsant, and muscle relaxant actions.

GABAergic synapses undergo short- and long-term plastic changes at both pre- and post-synapses. Postsynaptic plasticity mainly relies on the modulation of GABA_A_ receptors. Calcium influx through voltage-gated calcium channels (VGCCs) acts as a second messenger of an electrical signal regulating many physiological events. In particular, calcium entering through L-type VGCCs can activate different signaling pathways that lead to alterations in GABA_A_ receptors.

Voltage-gated calcium channels open in response to membrane depolarization, allowing calcium flux according to its electrochemical gradient. There are multiple types of VGCC with characteristic physiological and pharmacological properties and different distributions. In particular, L-type VGCCs exhibit a high voltage activation, large single-channel conductance, and slow voltage-dependent inactivation [22]. They are up-regulated by cyclic AMP (cAMP)-dependent phosphorylation mechanisms and antagonized by dihydropyridines, phenylalkylamines, and benzothiazepines. L-type and T-type VGCCs are expressed in many different cell types, whereas N-, P-, Q-, and R-type VGCCs are mainly present in neurons [22,23].

**Figure 1:**
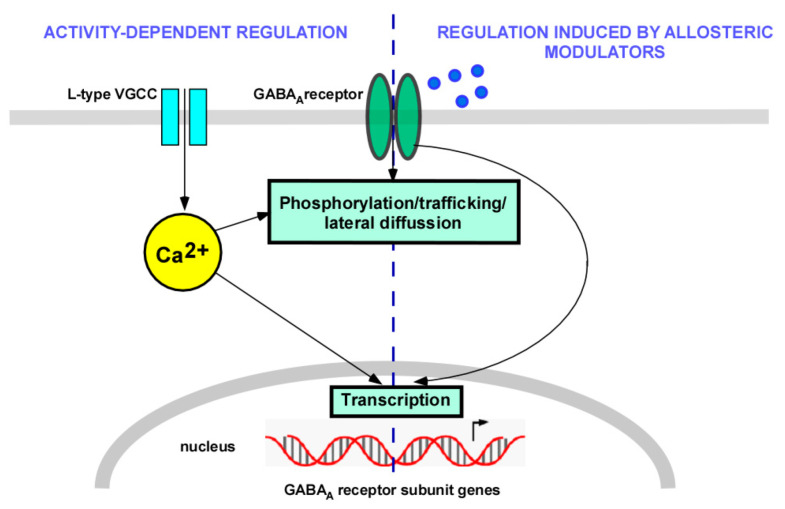
Regulation of GABA_A_ receptors by calcium and allosteric modulators. Calcium entering through L-type voltage-gated calcium channels (VGCCs) as a consequence of membrane depolarization can regulate GABA_A_ receptors’ function by means of changes in transcription, phosphorylation, trafficking, and lateral diffusion of receptors on the plasma membrane. These processes can also be regulated by the prolonged exposure of receptors to different allosteric modulators.

Voltage-gated calcium channels are composed of a large pore-forming α1 subunit and, with the exception of T-type VGCCs, are associated with auxiliary subunits named α2δ and β. An additional γ subunit has been identified as part of skeletal muscle VGCCs. There are ten isoforms of the α1 subunit, which can be classified into the following three families: Cav1, Cav2, and Cav3. These subunits primarily define the different types of calcium currents. There are four isoforms of α2δ and four isoforms of β subunits. Alternative splicing of many of these subunits increases the variability [23,24]. The Cav1 type of the α1 subunit is a part of the L-type VGCCs. Most L-type VGCCs in the brain (90%) are Cav1.2, whereas Cav1.3 channels represent 10%. Both isoforms are frequently present in the same cell. These channels regulate neuronal firing and activate signaling cascades involved in excitation–transcription coupling in the brain [22,24]. This review discusses different signaling pathways activated by the calcium influx through L-type VGCCs that control GABA_A_ receptor function by means of the regulation of receptor transcription, phosphorylation, intracellular trafficking, and synaptic clustering. These plastic mechanisms are crucial for the regulation of the interplay of synaptic excitation and inhibition that controls the function of neuronal circuits in the healthy brain (Figure 1).

## 2. Transcriptional Regulation

Activity-regulated gene expression in the central nervous system is part of a regulatory mechanism of neurons to respond to environmental stimulation, and is important in development, learning and memory, programmed cell death, and drug addiction [25]. This coupling of synaptic activity to the nucleus allows for the control of gene expression by promoting new transcription and epigenetic modifications.

The basal levels of free cytoplasmic calcium in neurons are actively maintained low (10–100 nM), relative to the extracellular milieu or intracellular storage compartments (2 mM), by pumping calcium into the internal stores or outside the cell. Therefore, neurons can rapidly sense and respond to an increase in the intracellular calcium concentration. Calcium can enter into the cytoplasm of a postsynaptic neuron in different ways: from the extracellular space through NMDA or AMPA receptors, VGCCs, or from internal stores. Interestingly, different responses are induced depending on the route of calcium entry, thus leading to multiple patterns of gene expression [26]. For example, in cortical neurons, calcium influx through L-type VGCCs or NMDA receptors differentially regulates the expression of brain-derived neurotrophic factor (BDNF), a neurotrophin that plays important roles during neuronal development and in synaptic plasticity [27]. These multiple responses may be the result of differences in the calcium channels in terms of gating properties, subcellular localization, and/or association with specific signaling molecules.

Because of several properties of L-type VGCCs, the calcium influx through these channels plays a predominant role in the coupling of the synaptic activity with transcription. First, L-type VGCCs are located in cell bodies and proximal dendrites, in relative proximity to the nucleus. Second, these channels have a large single channel conductance and a slow inactivation rate. Finally, they are physically associated with key signaling molecules important for transcriptional control [28].

Calcium entering through L-VGCCs as a consequence of membrane depolarization is sensed by a high-affinity binding protein, calmodulin, which is anchored to the α1 subunit of this channel. Calcium-bound calmodulin either stimulates—or is necessary to trigger—multiple cascades such as the activation of the guanine nucleotide exchange factor RasGRF, which then activates the Ras-mitogen-associated protein kinase (MAPK) signaling pathway, calcium/calmodulin-dependent protein kinases (CaMKs), adenylyl cyclase synthesis of cAMP and activation of protein kinase A (PKA), and the calcineurin-mediated pathway. In addition, a direct increase in calcium in the nucleus can be evoked by synaptic activity. The stimulation of these pathways results in the activation of multiple preexisting transcription factors in the nucleus by means of posttranslational protein modifications. One of the best-characterized calcium-regulated transcription factors is the cAMP response element (CRE)-binding protein (CREB). The activity of CREB is differentially regulated by phosphorylation at multiple sites. The calcium-activated transcription factors bind to the regulatory regions of genes to regulate their expression. Calmodulin is also anchored to the NR1 subunit of NMDA receptors, sensing the calcium that enters through these receptors [28,29,30].

The chromosomal localization and structure of the GABA_A_ receptor subunit genes, including the binding sites for transcription factors and regulatory elements, have been extensively studied [31,32]. Numerous evidence indicates that several neuronal activity-regulated transcription factors, such as CREB, nuclear respiratory factor 1 (NRF-1), specificity protein 4 (Sp4), and nuclear factor of activated T cell 4 (NFATc4), modulate the gene expression of GABA_A_ receptor subunits, suggesting a role of calcium signaling to the nucleus. The promoters of different GABA_A_ receptor subunit genes exhibit consensus binding sites for CREB, the typical calcium-dependent modulator of gene expression [31,32]. In fact, CREB positively regulates the transcription of the GABA_A_ receptor α1 subunit gene via a protein kinase C (PKC)-dependent mechanism [33] in rat cortical neurons. It has been reported that neuronal depolarization induces NRF-1 in rat cortical neurons, which stimulates the transcription of the GABA_A_ receptor β1 subunit gene [34], and Sp4 in murine visual cortical neurons, which stimulates GABA_A_ receptor α1/α2 subunit genes [35]. A positive excitatory feedback mechanism was demonstrated in adult mouse hippocampal stem/progenitor cells, in which the stimulation of GABA_A_ receptors induces neuronal depolarization that produces an enhancement in the intracellular calcium concentration. This increase results in the activation of NFATc4 via calcineurin, which in turn up-regulates GABA_A_ α2 and α4 subunit genes [36].

Immediate-early genes, which are downstream targets of CREB, further control GABA_A_ receptor subunit transcription, suggesting an indirect regulation of receptor expression by VGCCs. For example, early growth response factors (Egr) regulate the transcription of GABA_A_ subunit genes. In fact, Egr-1 up-regulates the expression of GABA_A_ receptor α2, α4, and θ subunit genes in the mouse hippocampus [37]. In addition, Egr-3 up-regulates the transcription of the GABA_A_ α4 subunit gene in the murine hippocampus after status epilepticus [38] and as a consequence of neurosteroid withdrawal [39]. The role of BDNF (the most widely investigated target of CREB) in mediating the activity-induced regulation of GABAergic transmission has been extensively documented [40,41]. The signaling pathway triggered by the precursor of BDNF, proBDNF, through binding to the p75 neurotrophin receptor (p75NTR), activates the Janus kinase (JAK)/signal transducer and activator of transcription (STAT) cascade, which leads to the induction of the inducible cAMP early repressor (ICER), resulting in transcriptional repression of the GABA_A_ receptor β3 subunit gene in the rat hippocampus [42]. During status epilepticus, the levels of BDNF increase, leading to a decrease in the expression of the GABA_A_ receptor α1 subunit and an increase in the expression of α4 subunit in the rat hippocampus. The transcriptional repression of the α1 gene occurs via the activation of the JAK/STAT signaling pathway, which produces an increase in the expression of ICER [43]. The transcriptional stimulation of the α4 gene is produced via a PKC/MAPK pathway that induces the synthesis of Egr3 [44]. On the other hand, during development, depolarization induced by the activation of GABA_A_ receptors leads to an increase in calcium influx through L-type VGCCs in rat cerebrocortical neurons, which stimulates the release of BDNF [45].

Numerous studies have reported that long-term exposure of GABA_A_ receptors to agonist or different modulators induces selective changes in the transcript levels of receptor subunits, suggesting a transcriptional regulatory process [46,47,48,49]. Although the signaling pathways triggered by the sustained receptor stimulation are not completely understood, the activation of L-type VGCCs seems to be involved. Chronic GABA exposure of rat primary cultured cortical neurons has been reported to induce a decrease in the GABA_A_ receptor density [50,51], which is contingent on the activation of L-type VGCCs and involves the selective inhibition of subunit gene transcription [51,52,53]. Different reports indicate that sustained treatments with benzodiazepines induce alterations in GABA_A_ receptors by mechanisms involving the activity of L-type VGCCs. L-type VGCC inhibitors prevented the reduction of GABA_A_ receptor currents produced by chronic benzodiazepine administration in the rat hippocampus [54]. Moreover, the transcriptional repression of the GABA_A_ receptor α1 subunit gene, induced by prolonged benzodiazepine treatment in rat cerebrocortical neurons, is contingent on the activity of L-type VGCCs [55]. As calcium influx through these channels can lead to the activation of PKA [28,29,30], and it has been shown that the activation of this kinase results in transcriptional inhibition of the α1 gene [33], it is tempting to speculate that chronic treatments with benzodiazepines result in the activation of a cascade that involves calcium influx through L-type VGCCs, which in turn activates a PKA signaling pathway, leading to transcriptional down-regulation of the GABA_A_ receptor α1 subunit gene. Interestingly, prolonged exposure of GABA_A_ receptors to benzodiazepines and ethanol regulates L-type VGCCs [56]. In particular, the chronic administration of benzodiazepines potentiates calcium currents through L-type VGCCs in rat hippocampal CA1 pyramidal neurons [57] and mouse cerebrocortical neurons [58], likely due to an increase in the expression of L-type VGCC Cav1.2, Cav1.3, and α2/δ1 subunits [58].

The role of activation of L-type VGCCs in the regulation of GABA_A_ receptor expression under certain pathological conditions, such as hypoxia, has been reported. Hypoxia has severe dysfunctional effects in the central nervous system associated with an imbalance between excitatory and inhibitory transmission, which leads to excitotoxicity [59]. Transient hypoxia reduces GABA_A_ receptor function in rat cerebrocortical neurons by a mechanism that depends on the calcium influx through L-type VGCCs [60]. This downregulation is accompanied by a decrease in the transcript levels of α1, α5, β2, and γ2 GABA_A_ receptor subunits in NT2-N neurons [61], suggesting a transcriptional regulation. Interestingly, hypoxia stimulates calcium entry through L-type VGCC, which activates calcineurin, resulting in a transient increase in L-type VGCC currents in cerebrocortical neurons. These results suggest the existence of a positive feedback loop that amplifies calcium signaling [62].

## 3. Trafficking Regulation

It was shown that distinct firing patterns of rat cortical pyramidal neurons, depending on the behavioral state, bidirectionally regulate the trafficking of postsynaptic GABA_A_ receptors by activating different subtypes of VGCCs. During wakefulness, the repetitive firing of these neurons induces the activation of L-type VGCCs, which produces the stimulation of GABA_A_ receptor endocytosis, finally resulting in long-lasting depression of somatic inhibition. In contrast, during slow-wave sleep, the slow membrane potential oscillation with firing at the depolarizing phase activates R-type VGCCs in addition to L-type VGCCs. The activation of R-type VGCCs stimulates GABA_A_ receptor exocytosis at a greater degree than the internalization mediated by L-type VGCC activation, leading to the long-lasting potentiation of somatic inhibition [63]. It remains unknown if these effects are mediated by changes in the phosphorylation state of GABA_A_ receptors.

The strength of GABAergic neurotransmission is contingent upon the degree of GABA_A_ receptor clustering that determines the number of receptors at synapses. Chronic changes in neuronal activity can lead to alterations in the number of synaptic GABA_A_ receptors, a phenomenon known as homeostatic synaptic plasticity. Saliba et al. [64] showed that a chronic blockade of L-type VGCCs in rat hippocampal neurons decreases the number of GABA_A_ receptors at the synapses, in parallel with a reduction in the amplitude of mIPSCs. Their results also indicate that calcium influx through L-type VGCCs regulates the insertion of newly translated GABA_A_ receptor subunits into the plasma membrane by a mechanism that involves the activity of the proteasome. These observations suggest a role of L-type VGCCs mediating adaptive homeostatic alterations in the strength of GABAergic neurotransmission in response to prolonged changes in neuronal activity.

## 4. Phosphorylation Regulation

Changes in the phosphorylation state of GABA_A_ receptors can alter the channel function, receptor trafficking to and from the cell surface, and their sensitivity to allosteric modulators [65,66]. In particular, GABA_A_ receptor subunits are substrates of different calcium-dependent protein kinases such as PKA and CaMKII, and thereby, VGCCs can play a role in the posttranslational regulation of this receptor.

### 4.1. PKA

The regulation of GABA_A_ receptor function by calcium-dependent kinases has been studied in both expression systems and neuronal environments. Experiments performed in heterologous expression systems showed that PKA-induced phosphorylation of β1 and β3 GABA_A_ receptor subunits differentially regulated receptor activity. Phosphorylation of β1 at S409 by PKA resulted in the inhibition of GABA responses, whereas phosphorylation of β3 at S408 and S409 by PKA led to the potentiation of GABA currents. Receptors containing β2 subunits were not phosphorylated by PKA [67,68]. The effect of PKA activation on GABA_A_ receptor function in the brain is complex, probably due to the coexistence of different populations of receptors with distinct subunit compositions [65].

In a recent study, Nakamura et al. [69] identified two phosphorylation sites on S359 and S379 of the α2 subunit in GABA_A_ receptors. The phosphorylation state of S359 depends on the activities of PKA and protein phosphatase 1 (PP1) and/or protein phosphatase 2A (PP2A), and regulates the binding of gephyrin and collybistin to α2 in hippocampal neurons. Therefore, the phosphoregulation of α2 is important to control the density of GABA_A_ receptors at inhibitory synapses.

Bohnsack et al. [70] demonstrated that the activation of PKA in cerebrocortical cultures led to a decrease in the expression of synaptic GABA_A_ receptor α4 subunits that was associated with an increase in the phosphorylation at S408/S409 of GABA_A_ receptor β3 subunits. In contrast, the activation of PKA resulted in an increase in the expression of extrasynaptic α4-containing receptors. As the total expression of α4 subunits remained unchanged, these alterations were likely due to changes in receptor trafficking.

The regulation of GABA_A_ receptors by allosteric modulators may be altered by the activity of calcium-dependent kinases. On the other hand, allosteric modulators can regulate GABA_A_ receptors by mechanisms that depend on the activation of these kinases. The inhibition of PKA reduced the sensitivity of GABA_A_ receptors to neurosteroids in hippocampal CA1 pyramidal neurons, suggesting that the action of this endogenous modulator depends on the phosphorylation status of this receptor [71]. Different reports demonstrated that the regulation of GABA_A_ receptors by continuous exposure to allosteric modulators is mediated through mechanisms that involve the activity of PKA. For example, the application of ethanol to cultured neurons altered GABA_A_ receptor trafficking, leading to changes in the expression of GABA_A_ receptor α1 and α4 subunits at the cell surface by a PKA-dependent mechanism [72,73,74,75]. In addition, the decrease in the amplitude of GABA_A_ receptor miniature inhibitory postsynaptic currents (mIPSCs) in CA1 pyramidal neurons, induced by a chronic treatment with flurazepam, was associated with a reduction in the PKA activity and PKA RIIβ protein [76]. A report from Ali et al. [77] showed that the uncoupling of the GABA/benzodiazepine site interaction produced by diazepam exposure of recombinant GABA_A_ receptors expressed in Sf9 cells was inhibited by the activation of PKA.

### 4.2. CaMKII

The activity of CaMKII enhanced GABA-evoked currents in a neuroblastoma-glioma hybrid cell line expressing α1β3γ2 GABA_A_ receptors by means of direct phosphorylation of S383 on β3 subunits. The activation of CaMKII seemed to also induce the phosphorylation of Y365/Y367 on γ2 subunits by endogenous tyrosine kinases. However, CaMKII failed to modulate the function of β2-containing GABA_A_ receptors expressed in this cell line [78,79]. In contrast, in cerebellar granule neurons, CaMKII modulated both β2- and β3-containing receptors [80]. CaMKII increased the amplitude and decay times of spontaneous inhibitory postsynaptic currents (sIPSCs) through a differential modulation of GABA_A_ receptors containing β2 or β3 subunits. Thus, the increased IPSC amplitude was associated with β2 GABA_A_ receptors and the prolongation of IPSC duration due to phosphorylation of the β3 GABA_A_ receptors [80].

The regulation of GABA_A_ receptors by orexins (neuropeptides involved in the stabilization of the waking state) seems to involve the activation of PKC and CaMKII. The exposure of HEK cells expressing α1β1γ2S GABA_A_ receptors and orexin receptors 1 (OX_1_R) to orexin-A inhibits GABA_A_ receptor currents. This effect depends on calcium and is associated with the phosphorylation of S409 on β1 subunits of GABA_A_ receptors by PKC and CaMKII. This crosstalk between orexins and GABA_A_ receptors could be relevant to the regulation of the sleep−wake cycle [81].

Postsynaptic depolarization of Purkinje cells can induce a long-lasting potentiation of GABAergic transmission at synapses formed by stellate cells on the Purkinje cells, referred to as rebound potentiation, which seems to be involved in motor learning [82,83,84,85,86]. Rebound potentiation is triggered by an increase in intracellular calcium as a result of VGCC activation. The induction of rebound potentiation depends on the activity of CaMKII [87], and the association between GABA_A_ receptor associated protein (GABARAP) and the GABA_A_ receptor γ2 subunit [88]. This form of plasticity is suppressed by the activation of GABA_B_ receptors during postsynaptic depolarization by means of a reduction of PKA activity [83,89]. This down-regulation of PKA activity leads to a decrease in the amount of phosphorylated dopamine- and cAMP-regulated phosphor-protein 32 kDa (DARPP-32). As phosphorylated DARPP-32 inhibits PP1, which in turn dephosphorylates CaMKII, the activation of GABA_B_ receptors results in the inhibition of CaMKII. A role for phosphorylation of GABA_A_ receptor β subunits by CaMKII in rebound potentiation is possible. In addition, the increase in the intracellular calcium concentration activates calcineurin, which dephosphorylates DARPP-32 and thus suppresses rebound potentiation [89].

The insertion of GABA_A_ receptors into the cell surface is regulated by a phosphorylation mechanism that involves the activation of L-type VGCCs. It was demonstrated in rat hippocampal cultures that neuronal activity leads to the accumulation of GABA_A_ receptors composed of α5/β3 subunits at the plasma membrane, leading to the stimulation of tonic currents, without influencing receptor endocytosis. This process is mediated by calcium influx through L-type VGCCs, which induces an increase in β3 S383 phosphorylation by CaMKII [90].

The effect of allosteric modulators of GABA_A_ receptors can be regulated by the activity of CaMKII. In fact, the stimulation of CaMKII in synaptosomal preparations induced an enhancement of benzodiazepine binding that was accompanied by an increase in the phosphorylation of the GABA_A_ receptor α1 subunit [91].

## 5. Regulation of Clustering/Lateral Diffusion

As the endocytosis and exocytosis of GABA_A_ receptors occur at extrasynaptic sites [92], the lateral diffusion of receptors within the plane of the plasma membrane represents a key process in targeting receptors to synapses. Therefore, the receptor exchange in and out of synapses by lateral mobility, and the receptor stabilization at synapses, are important steps that control synaptic strength. The diffusion properties of GABA_A_ receptors are regulated by their interaction with the scaffolding protein gephyrin. It was shown that synaptic GABA_A_ receptors have lower levels of lateral mobility than their extrasynaptic counterparts, and that gephyrin plays a role limiting the diffusion of receptors that favors their clustering at synapses [93].

Different reports demonstrate that clustering and lateral diffusion of GABA_A_ receptors can be regulated by calcium influx through L-type VGCCs. At early postnatal stages, depolarizing GABA actions induce gephyrin puncta in dendrites of mouse cortical pyramidal neurons via the activation of GABA_A_ receptors and L-type VGCCs [94]. The activity-dependent regulation of GABA_A_ receptor diffusion dynamics is also mediated by calcium influx. Bannai et al. [95] demonstrated that an enhanced excitatory synaptic activity in rat hippocampal neurons leads to a reduction in the cluster size of GABA_A_ receptors and a reduction in GABAergic mIPSCs. These changes occur in parallel with an enhancement of the diffusion coefficient of GABA_A_ receptors, which depends on the calcium signaling via calcineurin. The sources of calcium are NMDA receptors and L-type VGCCs [95]. Moreover, the diffusion properties of GABA_A_ receptors at the axon initial segment are regulated by an activity-dependent mechanism that involves the activation of calcium channels. It has been reported that the chronic depolarization of rat hippocampal neurons increases the diffusion of GABA_A_ receptors and decreases their synaptic residency time in the axon initial segment. This enhanced lateral mobility of receptors is induced by the activation of L-type VGCCs [96].

Interestingly, a report from Geisler et al. [97] indicated that presynaptic overexpression of individual α2δ-2 subunits of VGCCs induced an increase in postsynaptic GABA_A_ receptor clustering in cultured mouse hippocampal neurons. Surprisingly, this effect was observed in both GABAergic and glutamatergic synapses. The latter condition resulted in the formation of mismatched synapses in excitatory neurons, which may be associated with neuropsychiatric disorders.

## 6. Regulation of GABA_A_ Receptors by Other Sources of Calcium

Numerous evidence indicates that an increase in cytosolic calcium induced by its influx through NMDA receptors or release from intracellular stores can regulate GABA_A_ receptor function. Calcium influx via NMDA receptors can mediate long-term plasticity of GABAergic transmission by changes in GABA_A_ receptor trafficking or lateral diffusion. The activation of NMDA receptors can induce inhibitory long-term potentiation (iLTP) or depression (iLTD), depending on the degree of receptor activation, which leads to different levels of intracellular calcium increase. Moderate activation of NMDA receptors that results in a moderate intracellular calcium rise induces iLTP by means of the translocation of CaMKII to inhibitory synapses, which phosphorylates GABA_A_ receptor β3 subunits at serine 383, producing an increase in the insertion and stabilization of receptors at synapses [98,99,100,101]. In contrast, the strong activation of NMDA receptors induces higher intracellular calcium levels, which elicit iLTD. The mechanism of iLTD involves the dephosphorylation of the GABA_A_ receptor γ2 subunit at serine 327 by calcineurin inducing an enhancement of lateral mobility of GABA_A_ receptors, which causes the dispersal of synaptic receptors [95,101,102,103].

Calcium release from intracellular stores has been demonstrated to regulate the lateral mobility of GABA_A_ receptors on the plasma membrane. Bannai et al. [95,104] reported a mechanism of homeostatic stabilization of GABAergic synapses in rat hippocampal neurons that occurs under conditions of basal synaptic activity and involves calcium release from intracellular stores. The activation of group I metabotropic glutamate receptors by ambient glutamate stimulates IP3 receptor-dependent calcium release from the endoplasmic reticulum, which in turn activates PKC, leading to a constraint of GABA_A_ receptor lateral diffusion and an increase in receptor clustering. The results from Brady et al. [105] showed that during the transition from depolarizing, excitatory GABA_A_ receptor signaling to a hyperpolarizing inhibitory response, a phase with depolarizing inhibitory receptor activity occurred in the cortical neurons. During this period, the activity of GABA_A_ receptors induced plastic changes in GABAergic synapses, which depend on intracellular calcium release, extracellular signal-regulated kinase (ERK), and BDNF. The exposure of cortical neurons to muscimol decreased the synaptic localization of γ2-containing GABA_A_ receptors and gephyrin. These changes were associated with a reduction in synaptic currents and an increase in tonic currents mediated by γ2 GABA_A_ receptors. The dispersal of γ2 GABA_A_ receptors was mediated by ERK and BDNF signaling, whereas the synaptic gephyrin declustering was regulated by ERK signaling alone. The activation of ERK depended on the release of calcium from intracellular stores. In addition, muscimol treatment induced a reduction in presynaptic GAD65 levels by a mechanism that involved the activation of BDNF/TrkB signaling.

Several studies indicate that GABA_A_ receptor trafficking can be down-regulated under pathological or pharmacological conditions by changes in the intracellular calcium that result from the activity of NMDA receptors or the calcium mobilization from or into intracellular stores. During prolonged epileptiform activity, in an in vitro model of *status epilepticus* in rat hippocampal neurons, calcium entry through NMDA receptors was shown to activate calcineurin producing a reduction in the surface levels of GABA_A_ receptors composed of α2 subunits [106]. Changes in calcium concentration in the endoplasmic reticulum were demonstrated to regulate GABA_A_ receptor trafficking. The D219N mutation in the GABA_A_ receptor α1 subunit, which is associated with an idiopathic epilepsy, leads to the retention of the subunit in the endoplasmic reticulum and to its excessive degradation by the proteasome. Verapamil, an L-type VGCC blocker, increases calcium concentration in the endoplasmic reticulum facilitating the trafficking of the mutant subunit to the plasma membrane in HEK293 and neuronal SH-SY5Y cells. It has been proposed that the increased calcium in the endoplasmic reticulum stimulates the interaction between the α1(D219N) subunit and two lectin chaperones, calnexin and calreticulin, which enhances the folding and assembly of the mutant subunit, enabling its forward trafficking [107]. Prolonged exposure of rat cerebrocortical neurons to diazepam induces the disassembly of GABAergic synapses, which is caused by a decrease in GABA_A_ receptor surface levels. The underlying mechanism involves PLCδ activation, calcium mobilization from intracellular stores, and the activation of calcineurin, which results in the dephosphorylation of γ2 GABA_A_ receptor subunits, finally leading to an increase in receptor internalization [108].

## 7. Conclusions and Future Directions

**Figure 2:**
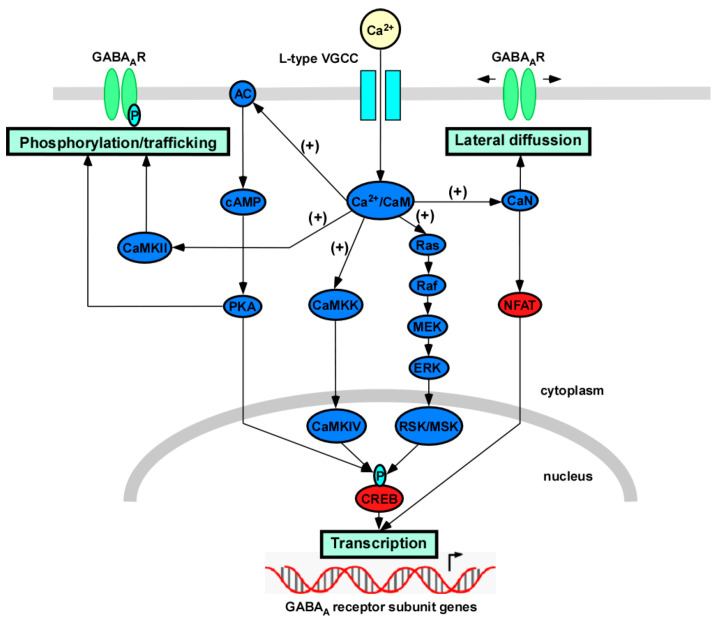
Different signaling mechanisms triggered by the activation of L-type VGCCs that lead to GABA_A_ receptor regulation. The calcium influx through L-type VGCCs activates different pathways, such as Ras-MAPK, CaMK, PKA, and calcineurin cascades, which mediate the activity-dependent regulation of transcription, phosphorylation, trafficking, and lateral diffusion of GABA_A_ receptors. The gene expression of different GABA_A_ receptor subunits is regulated by several calcium-dependent transcription factors, such as CREB and NFAT. Changes in the phosphorylation state of GABAA receptors by calcium-activated kinases modulate receptor function and trafficking. Lateral diffusion of GABAA receptors on the plasma membrane is regulated by calcium signaling via calcineurin. GABA_A_R—GABA_A_ receptor; AC—adenylyl cyclase; CaM—calmodulin; CaMKK—calcium/calmodulin-dependent protein kinase kinase; CaMKII—calcium/calmodulin-dependent protein kinase II; CaMKIV—calcium/calmodulin-dependent protein kinase IV; CaN—calcineurin; ERK—extracellular signal-regulated kinase; MEK—mitogen-activated protein kinase kinase; MSK—mitogen- and stress-activated protein kinase; Raf—Ras-activated factor; RSK—ribosomal s6 kinase.

The activation of L-type VGCCs triggers different signaling cascades that lead to changes in GABA_A_ receptor function (Figure 2).

L-type VGCCs play an important role in linking synaptic activity with transcription because of their gating properties, subcellular localization, and association with key signaling molecules. Calcium entering through L-type VGCCs as a consequence of membrane depolarization leads to the activation of multiple transcription factors in the nucleus. Several of these transcription factors bind to the promoters of different GABA_A_ receptor subunit genes regulating receptor expression. Prolonged stimulation of GABA_A_ receptors by positive allosteric modulators also regulates the transcription of specific GABA_A_ receptor subunit genes by a mechanism that involves calcium influx through L-type VGCCs.

Chronic changes in neuronal activity induce alterations in phosphorylation, intracellular trafficking, and lateral diffusion on the plasma membrane of GABA_A_ receptors that lead to changes in the GABA_A_ receptor number at postsynapses by mechanisms that depend on the activation of L-type VGCCs. Therefore, the activity of L-type VGCCs plays an important role in regulating the strength of GABAergic neurotransmission in physiological, pharmacological, and pathological conditions.

**Figure 3:**
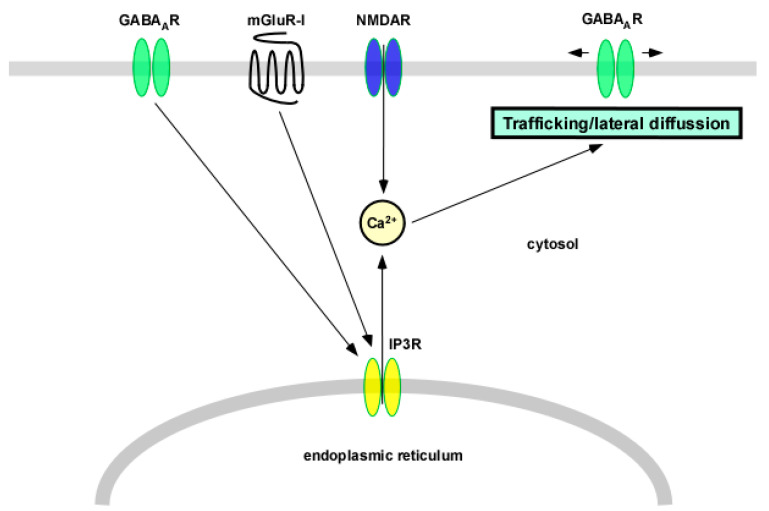
Regulation of GABA_A_ receptors by intracellular calcium from different sources other than the influx through L-type VGCCs. The increase in cytosolic calcium induced by the release from intracellular stores (because of the activation of group I metabotropic glutamate receptors or the stimulation of GABA_A_ receptors) or the influx through NMDA receptors regulates trafficking and lateral diffusion of GABA_A_ receptors. GABA_A_R—GABA_A_ receptor; mGluR-I—group I metabotropic glutamate receptors; NMDAR—NMDA receptor; IP3R—inositol trisphosphate receptor.

GABA_A_ receptors are also regulated by an increase in cytosolic calcium induced by its influx through NMDA receptors or release from intracellular stores (Figure 3).

The nervous system has the special property of continuously adapting to stimuli. For example, alterations in neuronal activity dynamically regulate synaptic function. Although activity-dependent regulation of synaptic strength was initially focused on excitatory synapses, increasing evidence indicates that GABAergic synapses also exhibit this type of plasticity. Calcium entering through VGCC represents a key messenger linking synaptic activity to the regulation of GABA_A_ receptor function. Despite the numerous studies on the mechanisms of the activity-dependent regulation of GABA_A_ receptors, important questions remain to be elucidated in future investigations, namely:

- To further investigate the physiological role of GABA_A_ receptor plasticity, more in vivo studies are required.
- Future experiments are needed to differentiate the specific mechanisms of GABA_A_ receptor regulation that occur during normal development from the ones that persist into adulthood.
- The plastic mechanisms of receptor regulation associated with neurodevelopmental disorders, such as epilepsy, autism, and schizophrenia, should be also investigated.
- As activity-dependent plasticity can occur simultaneously at inhibitory and excitatory synapses, it is important to study how these two processes are coordinated.

Considering the pharmacological importance of GABA_A_ receptors, the elucidation of the molecular bases of receptor regulation will be crucial in the search for new therapeutic strategies.

## Data Availability

Not applicable.
